# Managing HCV infection: present and future

**DOI:** 10.1186/1471-2334-14-S2-S5

**Published:** 2014-05-23

**Authors:** Jürgen K Rockstroh

**Affiliations:** 1 Department of Medicine I, Bonn University Hospital, Bonn, Germany

## 

The development of direct acting antiviral agents against the hepatitis C virus has revolutionized treatment paradigms for hepatitis C. In 2011 the first HCV protease inhibitors boceprevir and telaprevir were approved, which still needed to be combined with pegylated interferon and ribavirin and were only usable in patients with genotype 1. However, the new triple therapy achieved successful cure rates of hepatitis C in almost 70% of treatment naïve patients and immediately became the new gold standard of HCV therapy. With the development of further agents, in particular the recent licensing of sofosbuvir and simeprevir, two further DAA’s have become available. Thereby, for the first time Interferon free regimens are already available for genotype 2 and 3 patients allowing cure rates approaching 90% after 12 weeks of sofosbuvir and ribavirin for genotype 2 treatment naïve as well as treatment-experienced patients and 24 weeks of all oral therapy for patients with genotype 3 (again treatment-naïve or experienced). For genotype 1 the combination of sofosbuvir with pegylated interferon and ribavirin allows curing over 90% of patients after 12 weeks of triple therapy and therefore has become the preferred HCV therapy for this particular patient group. Also combination of sofosbuvir with other agents have been presented in particular simeprevir or daclatasvir leading to high success rates above 90% even in patients who failed previous triple therapy with one of the first HCV protease inhibitors. Therefore, where available, 2 DAA combinations ± ribavirin are rapidly becoming the new HCV treatment standard for more difficult to treat patient groups. In the near future combinations of antiviral agents with no cross resistance and improved safety profile will further reduce treatment durations and will become the new recommended HCV regimens making HCV the first viral infection to be possibly eradicated worldwide.

